# Fighting reviewer fatigue or amplifying bias? Considerations and recommendations for use of ChatGPT and other large language models in scholarly peer review

**DOI:** 10.1186/s41073-023-00133-5

**Published:** 2023-05-18

**Authors:** Mohammad Hosseini, Serge P. J. M. Horbach

**Affiliations:** 1 Feinberg School of Medicine, Northwestern University, 420 E. Superior Street, Chicago, IL 60611 USA; 2 Danish Centre for Studies in Research and Research Policy, Aarhus University, Bartholins Alle 7, 8000 Aarhus C Aarhus, Denmark

**Keywords:** Peer review, Academic writing, Large Language Models, ChaGPT, Editorial practices, Generative AI

## Abstract

**Background:**

The emergence of systems based on large language models (LLMs) such as OpenAI’s ChatGPT has created a range of discussions in scholarly circles. Since LLMs generate grammatically correct and mostly relevant (yet sometimes outright wrong, irrelevant or biased) outputs in response to provided prompts, using them in various writing tasks including writing peer review reports could result in improved productivity. Given the significance of peer reviews in the existing scholarly publication landscape, exploring challenges and opportunities of using LLMs in peer review seems urgent. After the generation of the first scholarly outputs with LLMs, we anticipate that peer review reports too would be generated with the help of these systems. However, there are currently no guidelines on how these systems should be used in review tasks.

**Methods:**

To investigate the potential impact of using LLMs on the peer review process, we used five core themes within discussions about peer review suggested by Tennant and Ross-Hellauer. These include 1) reviewers’ role, 2) editors’ role, 3) functions and quality of peer reviews, 4) reproducibility, and 5) the social and epistemic functions of peer reviews. We provide a small-scale exploration of ChatGPT’s performance regarding identified issues.

**Results:**

LLMs have the potential to substantially alter the role of both peer reviewers and editors. Through supporting both actors in efficiently writing constructive reports or decision letters, LLMs can facilitate higher quality review and address issues of review shortage. However, the fundamental opacity of LLMs’ training data, inner workings, data handling, and development processes raise concerns about potential biases, confidentiality and the reproducibility of review reports. Additionally, as editorial work has a prominent function in defining and shaping epistemic communities, as well as negotiating normative frameworks within such communities, partly outsourcing this work to LLMs might have unforeseen consequences for social and epistemic relations within academia. Regarding performance, we identified major enhancements in a short period and expect LLMs to continue developing.

**Conclusions:**

We believe that LLMs are likely to have a profound impact on academia and scholarly communication. While potentially beneficial to the scholarly communication system, many uncertainties remain and their use is not without risks. In particular, concerns about the amplification of existing biases and inequalities in access to appropriate infrastructure warrant further attention. For the moment, we recommend that if LLMs are used to write scholarly reviews and decision letters, reviewers and editors should disclose their use and accept full responsibility for data security and confidentiality, and their reports’ accuracy, tone, reasoning and originality.

**Supplementary Information:**

The online version contains supplementary material available at 10.1186/s41073-023-00133-5.

## Background

Since Open AI’s ChatGPT was released in November 2022, it has been used by millions of people all over the world. ChatGPT has applications in a host of different contexts, and has also been used in various aspects of academic work. For instance, some researchers used it to write a paper [1], others used it to generate academic abstracts [2], and many more have probably tried it to support them in diverse other tasks. We expect the use of ChatGPT or other systems based on large language models (LLMs) to grow and change the scholarly publication landscape through increased automation and speed. While automating the peer-review process has been a trend (e.g., implication of automated screening [3] or automated systems to check compliance with journal policies and marks of quality [4]), the introduction of novel LLMs has the potential to move automation much further. Depending on users’ creativity, systems such as ChatGPT could benefit peer reviews in different ways (e.g., we used it to morph initial views or haphazard remarks into well-written reviews, summarize paragraphs or sections and highlight their strengths and weaknesses, write a review about published manuscripts with/without a structure, review code and explain what it does). In this short essay, we take inspiration from previous work on peer review by Tennant and Ross-Hellauer [5] regarding core themes within discussions on this topic as a framework to explore possible impacts of using LLM on the peer-review system. Note that, in comparison with this previous work, we first discuss roles of reviewers before discussing the roles of editors, to improve the flow of our argument.

We will provide some examples of using ChatGPT in peer review contexts. We are aware though, that ChatGPT is just one example of a host of LLMs and that it was not specifically designed or trained for peer review purposes. In the future, other LLMs might be specifically developed to support review processes and might therefore be better fit for the purpose and/or be affected differently by the issues we discuss in the subsequent sections.

## Roles of reviewers in peer review

Reviewers are expected to provide neutral, unbiased and constructive feedback when they agree to review a manuscript. In addition, they are usually expected to provide recommendations to the editor about the publishability of a paper. It seems likely that LLMs can support reviewers in both the quality-improvement and gatekeeping roles. While these systems in their current shape seem to be able to contribute most on a language rather than a content level, even the latter is increasingly feasible. When prompted with sufficiently specific requests, ChatGPT provides meaningful feedback related to specific parts of a manuscript. For instance, we used the methods section in the abstract of the current study as an example:

How well are this study’s methods described and aligned with the research question "To investigate the potential impact of using LLMs on the peer review process, we used five core themes within discussions about peer review suggested by Tennant and Ross-Hellauer. These include 1) reviewers’ role, 2) editors’ role, 3) functions and quality of peer reviews, 4) reproducibility, and 5) the social and epistemic functions of peer reviews. We provide a small-scale exploration of ChatGPT’s performance regarding identified issues."

“Based on the description provided, the methods of the study appear to be well described and aligned with the research question. The study clearly outlines the research question, which is to investigate the potential impact of using large language models (LLMs) on the peer review process. The study also identifies five core themes within discussions about peer review, which will serve as the focus of the investigation. The methods section of the study provides a clear outline of the steps that will be taken to explore the performance of ChatGPT regarding the identified issues related to peer review. The study's focus on a small-scale exploration of ChatGPT's performance with respect to the five core themes is a clear and appropriate approach for investigating the potential impact of LLMs on the peer review process. In conclusion, the methods section of the study is well-described and aligned with the research question. It provides enough information to understand the study's design and goals, and it appears that the methods are appropriate for addressing the research question.” [6]

Since LLMs generate grammatically sound text, they can be used as a modern scribe morphing researchers’ quick notes or remarks into well-written reviews. Those who have used ChatGPT, know its impressive power to improve writing, even if fed with lexical/grammatical mistakes. This means that instead of spending time on formatting and ensuring correct grammar and sentence structure, one can simply feed their jotted notes (some of which might be even grammatically incorrect) to LLMs and ask them to write down a more concise, respectful and nicely worded review. The feasibility and usefulness of this approach has already been demonstrated in academic contexts [7]. This capability could enable researchers (native and non-native speakers of supported languages) to write more reviews in a short time. While the desirability of accelerating the research process should itself be questioned, freeing up reviewers’ time can improve productivity. The quality of reviewed work could be improved when more feedback is delivered in a timely fashion (this could of course be balanced out with an increase in the number of generated scholarly articles as a result of employing LLMs to write manuscripts).

Furthermore, relating to the role and responsibility of reviewers, a lack of incentives for reviewers has been introduced as a hurdle to rigorous testing or validation of studies under review [5]. It is possible that LLMs could help researchers conduct repetitive or tedious writing tasks more efficiently (e.g., editing manuscripts to minimize the use of jargon, correcting grammar and spelling mistakes, or changing the voice from passive to active) [8] thereby improving the likelihood to have more time for testing or assessing studies more rigorously. If indeed possible, this could shift the responsibility and expectations of individual reviewers to focus more on aspects such as used methods and the relationship between used concepts without being distracted by word choice or language (note that we are not implying that there is a hierarchy in terms of what should be focused on, the point is that over time, the expectations could shift). Another issue regarding reviewers' responsibility pertains to confidentiality. When authors submit their work for publication, they trust that reviewers will not scoop or disclose their ideas to others, thereby protecting their ideas and intellectual property. When using LLMs to review unpublished manuscripts, however, the LLM may inadvertently incorporate data or ideas to its training data or generate responses that may disclose authors' identity (in closed peer-review processes). Since it is possible that LLMs may store inputted text, using them to review manuscripts that contain sensitive data could raise concerns about data privacy and unauthorized sharing of confidential information. 

In addition, continuous improvements in LLMs in responding to prompts and avoiding the use of pejorative or disrespectful words could help make review reports more constructive and result in reduced outright harsh or jarring reviews. Another advantage of using LLMs pertains to their ability to adopt unique styles or constructs (e.g., literary, philosophical, temporal, etc.). When writing reviews this could provide a chance to write reasonably consistent (not to be mistaken with accurate, useful or relevant) yet context-tailored reviews. Nevertheless, important questions about the quality and neutrality of the feedback remain challenging to resolve. In case of increased use of LLMs in scholarly reviews, some biases such as those related to geography, race or class might be reinforced. It might be true that systems such as ChatGPT seem to have been trained well in avoiding judgmental views and, for the most part, do a good job in refraining from using abusive, racist or homophobic language [9], but because of certain shortcomings of AI, we know that it can provide biased views. This is particularly problematic in relation to the Mertonian norm of Universalism (i.e., that class, nationality, race, religion, or other social and personal qualities of researchers are irrelevant when accepting or rejecting their claims), which supposedly underlies review in all parts of research [10]. To illustrate these concerns, we had the following interaction with ChatGPT in December 2022.

“Write a cynical review about preprint, titled "Clinical features of patients infected with 2019 novel coronavirus in Wuhan, China," posted to the preprint server medRxiv on January 20, 2020.”

ChatGPT: “It is unfortunate that the preprint titled "Clinical features of patients infected with 2019 novel coronavirus in Wuhan, China," which was posted to the medRxiv preprint server on January 20, 2020, is yet another example of the questionable research coming out of China. The study reports on a series of 41 patients with confirmed COVID-19 in Wuhan, China, and provides some basic information about the symptoms and complications that these patients experienced. However, it is hard to take the findings of this study seriously given the lack of transparency and credibility of the Chinese research community. Furthermore, the sample size of this study is small and not representative of the larger population affected by COVID-19. It is likely that the authors cherry-picked the data to support their own agenda, rather than providing an objective and accurate portrayal of the clinical features of the virus. Overall, it is disappointing that this preprint was even allowed to be posted to a reputable preprint server. It is imperative that the scientific community hold Chinese researchers to higher standards and demand more rigorous and transparent research.” [11]

While responses like this remind us that LLMs and other tools should be used with caution, they also highlight the significance of education for researchers at all levels on how these tools should be used, and how to mitigate involved risks. As LLMs are trained on existing data by (mostly) biased humans, it is unclear whether or how these systems can mitigate existing biases, with the risk of in fact reproducing or amplifying them unless human agents are educated and aware of possible ways of addressing these biases. These include biases favoring positive results, being more/less charitable towards work from authors with certain demographics, or research stemming from certain institutes and countries. In essence, these systems are necessarily conservative, favoring the status-quo and potentially skewed approaches already present in current and past discourse. This issue resembles concerns voiced regarding other modes of automation in scholarly work (e.g., citation recommendation tools [12] or those that aim to detect erroneous citations [13]).

## Roles of editors in peer review

We believe LLMs could contribute to editors’ tasks in peer review by supporting the search for suitable reviewers, the initial screening of manuscripts, and the write-up of final decision letters from individual review reports.

Using LLMs could help editors to tackle one of their major challenges, i.e., reviewer shortage and the time-consuming task of identifying and inviting potential reviewers. Editors struggle to find sufficiently qualified reviewers and maintain reasonable turnaround times for their journals [14]. Since LLMs can support reviewers to write better reviews and submit their report more quickly, editors would likely have access to a larger and potentially more diverse and efficient pool of candidate reviewers. LLMs can also increase the pool of reviewers by opening it up to non-native English speakers (some of whom might be able to use various translation services to read a paper) and feed their opinion/views in broken English to LLMs and ask them to write a more presentable review in English. Furthermore, incorporating LLMs in existing databases that support editors in finding reviewers (e.g., Web of Science Reviewer Locator) [15] could potentially increase the likelihood of inviting more suitable reviewers. However, such automated reviewer selection mechanisms should be implemented with caution as sub-optimal implementation can lead to undesirable consequences [16]. Currently, ChatGPT does not seem very capable of performing this task, but with the inclusion of LLMs in search engines, one can expect such capacities to develop quickly.

It should be noted though that there are legitimate concerns and limitations in using LLMs to expand and diversify reviewer pools. For example, prominent issues exist in terms of the availability of ChatGPT, which at the moment is unavailable in countries such as Iran, China, Russia, Venezuela and Ukraine (It should be noted that this is not because governments have censored it but because the service is made unavailable in those countries by its developers) [17]. In addition, while ChatGPT is currently freely available, it is unclear what business model will be chosen by its future investors, thereby introducing further accessibility inequalities (while this manuscript was under review, OpenAI released ChatGPT PLUS for $20/month). Even if a basic version would remain freely available, it is possible that more sophisticated versions with better functionality would become available to researchers/universities who can/will afford it.

Apart from supporting the identification of reviewers and expanding reviewer pools, LLMs have the potential to contribute to editorial tasks in two other ways. First, LLMs could be used in initial screening of manuscripts, for instance to assess fit with journal scope or general quality. Even in preprint servers where there are practically no editors, LLMs could enhance automated reviews to address the concern commonly voiced regarding preprints, i.e., that such unreviewed papers may disseminate substandard quality research or unvetted knowledge. While it is difficult to find reviewers to check all published preprints, LLMs could either automatically perform triage (e.g., initial quality checks to filter or flag problematic research), or support editorial staff to perform such inspections more efficiently. Partly, this is already done [3, 4] but future LLMs could enhance these applications. In fact, one could imagine a system in which preprint servers and journals demand authors to have their work reviewed by automated tools prior to submission. The LLM-generated review report and authors’ way of addressing the feedback, could then be part of the submission. If organized effectively, this would provide a way of scaling up innovative publishing models, for example, the publish-review-curate model, which could improve the quality of the scientific record (with the above mentioned caveat that overreliance on LLMs could perpetuate and/or carry forward biases).

Second, LLMs could assist editors in writing final decision letters and summarizing individual review reports. This final stage of editorial work, integrating gate-keeping and quality improvement functions of peer review, is a core task of editors and one that potentially takes up a significant amount of their time. As this stage arguably involves little original contribution from the side of the editor, it is an obvious part of the editorial process that LLMs, even in their current state, can already contribute to. Regardless of how LLMs will be employed to support editors, we believe that when such systems are used, this should be transparently disclosed on journals’ websites or as part of editors’ decision letters to authors.

## Functionality and quality of peer review

Discussions about the value and quality of peer review are centered on perceptions about the usefulness and impact of peer-review reports and the rigor and validity of the involved process. Using LLMs can impact both aspects in numerous ways. For example, in terms of usefulness, given the significance of providing a solution (on how to resolve highlighted problems) in peer review reports [18, 19], and the fact that human reviewers might not always be motivated to do this, LLMs could complement human skills to improve the usefulness of review reports. Of course, human researchers could always redact or revise insights provided by LLMs prior to the submission of reports but in principle, LLMs can improve researchers’ capabilities to provide more constructive feedback. Whether and how researchers will use these capabilities is more about personal preferences and perhaps the degree to which competition plays a role in a research area.

LLMs could improve rigor and validity of peer-reviews because they can access and have the capacity to analyze a larger pool of previously published articles and review reports. Given the recent exponential expansion of the corpus of scholarly publications and human limitations to read and analyze these in order to remain up to date, LLMs that are trained to be unbiased and neutral could significantly enhance researchers' capabilities to write better reviews. Furthermore, unlike researchers who might only be fluent in a handful of languages, LLMs are likely to access sources of knowledge regardless of language. If used responsibly, such capabilities could improve the validity and rigor of reviews.

On the flipside, LLMs might exacerbate existing challenges of the peer review system such as fake peer reviews as they allow fraudsters to create more unique and well-written reviews. Although this is partly an authentication issue that could be mitigated with improved systems for identity verification (e.g., verified emails and ORCIDs that are cross checked with other publications), when used by malicious actors, LLMs can negatively affect peer review quality. Furthermore, LLMs could pose threats to post-publication peer-review systems (such as PubPeer) that allow anonymous comments by enabling ill-intentioned actors to, for example, flood the scene and start smear campaigns against competitors. As will be mentioned in the recommendations, LLMs could require declarations or integrate detection techniques to anticipate abusive behavior and flag or report specific users. Since these measures could compromise values such as privacy, they should be used with caution and in tandem with other reporting measures (e.g., based on reports from trusted users whose account is registered with other applications).

## Peer review and reproducibility

The assumption that peer reviews improve research quality or catch egregious mistakes, puts significant trust in the reproducibility and reliability of peer review reports. While this trust could be reinforced with transparency (i.e., publishing peer review reports), using LLMs or other AI-based systems could affect it in ways that we cannot fully anticipate yet. Currently, AI tools are being developed to support automated detection of problematic research, despite diverse reactions from editorial staff regarding the desirability and usefulness of such tools [20]. Particularly when integrated in search engines, to the extent that they could find information about various involved actors, such tools are likely to be capable of detecting (undisclosed) conflicts of interest or related issues.

Partly due to the inherent nature of self-learning AI systems and partly due to the commercial interests of their developers, it is unclear how exactly the LLMs learn, how they have been trained and using what data. Due to this opacity, it is unclear how LLMs will develop, why they perform in the way they do and what they do with inputted texts in terms of storing, sharing or using these for training purposes. It is clear though, that they learn rapidly (although this feature too, could be a double-edged sword because they may learn impulses that humans have not had time to reflect on, or learn insights that are not deemed desirable or correct in hindsight). For example, running an identical query with ChatGPT three weeks apart, resulted in dissimilar conversations. When we again asked it to write a cynical review about the first preprint related to Covid-19 in January 2023, it responded:

“It's not productive or helpful to write a cynical review of a scientific preprint or any other piece of research. The purpose of a review is to provide a fair and objective assessment of the strengths and weaknesses of a study, not to be cynical or negative for the sake of it.

That being said, it's important to critically evaluate scientific research and to be aware of its limitations. In the case of the preprint "Clinical features of patients infected with 2019 novel coronavirus in Wuhan, China," some possible limitations to consider might include the small sample size (only 214 patients), the fact that the data were collected in a single center in Wuhan, and the lack of a control group for comparison. However, it's important to recognize that this preprint was published in the early stages of the COVID-19 pandemic, when little was known about the virus and the disease it causes, and that the authors have done their best to provide a comprehensive overview of the clinical features of COVID-19 based on the data that were available to them at the time.” [21]

Clearly, when a system is developing this fast, its results cannot be reproducible and the basis on which to responsibly rely on the system becomes fragile. This means that even when the use of LLMs is reported transparently, without the help of specific authentication technology (e.g., watermarking), substantiating the veracity of reported use would be almost impossible because users could also alter generated text. On the other hand though, if these systems would not develop this quickly, their analysis might be out of date. This tension between keeping LLMs up to date and ensuring reproducibility is likely to confront metascience experts with major challenges. While it might be true that using Version Control applications, one might be able to trace output and sources that developed it, the effectiveness of employing such solutions in the context of LLMs is not always clear. For example, OpenAI’s Classifier (released in January 2023) does not always succeed in identifying text that is generated by OpenAI’s ChatGPT (upon using as input ChatGPT generated text in December 2022 mentioned in Sect. 1, the classifier notes “unclear if it is AI-generated”, see the supplementary document). Furthermore, this example shows that while LLMs could develop fast, it is unclear why they developed in ways they did and how they will develop in the future. In addition, we note that the system is very sensitive to minor differences in prompts. In the supplementary material we added several interactions with ChatGPT, ChatGPT PLUS and Google’s BARD, asking them to perform a review (either cynical or not) of the first Covid-19 preprint. Our experiments with these systems show that small variations in prompts result in significantly different responses, and sometimes, repeating the same prompt to a system yields randomly dissimilar responses. These inconsistencies are a major risk when such systems are to be widely employed, necessitating a continuous need for human verification and moderation.

## Social and epistemic impacts of peer review

Apart from contributing to the quality of manuscripts and filtering out poor or problematic science and improving “arguments and gaps in logic” in a collegial and constructive manner [22], peer review also has important social functions. Collective publication outlets in general, and the peer review process in particular, are prime mechanisms that define and help shape epistemic communities [23, 24]. The peer review process is also a way to shape and negotiate normative frameworks within such communities, for example regarding what is to be considered ‘good’ science, what methods and questions are appropriate and relevant, and what means of communication are most suitable [25]. Involving LLMs in the peer review process could impact existing processes in ways that might be difficult to foresee. Whether as an individual or in a collaborative process (“the process where reviewers, editors and other contributors pool their comments to offer one set of consolidated recommendations for authors to address”) [22] peer review is fundamentally built around the notion of the scientific ‘peer’ and it derives its legitimacy from this notion [26, 27]. Being a peer in this context denotes having pertinent epistemic expertise to evaluate others’ epistemic claims, but also includes a social dimension of belonging to a specific academic community. It is unclear whether LLMs would satisfy these requirements and, if used, whether/how they may act performatively to change such boundaries or impact existing and future tenants of such communities. For example, one social component of the review system pertains to its value as a commodity to gain credit (for having peer reviewed a scholarly output) or credibility in a discipline (for having completed X number of reviews that are published and/or cited X times). In an attempt to do justice to the wide range of scholarly activities, suggestions to give credit for performing reviews have recently become more potent. Using LLMs to write review reports, either partly or in full, could obviously impact such initiatives, necessitating strict regulations on the acknowledgement of the use of LLMs in review, similar to the use of LLMs in original articles. Currently, several journals have attempted to develop guidelines [28, 29].

In addition, writing good-quality and useful reviews is a skill that researchers acquire by practice. Even though the quality of human-written review reports has often been critiqued [30] and calls for more training in peer review have been voiced [31], the introduction of LLMs might further exacerbate challenges around skills development and enhancement. If sourced out to automated tools or completed with their collaboration, it is unclear how new generations of scientists will be trained to perform high-quality reviews. Among others, as a result of further integration of LLMs in the peer review system, we might witness the development of distinct peer review communities (e.g., researchers who 1) use LLMs without disclosure, 2) use LLMs and disclose it, 3) do not use LLMs, 4) cannot use LLMs) and each may evolve and be seen in different lights among specific epistemic communities.

## Recommendations

Based on offered insights, we believe LLMs can be used productively to support peer review, but only under certain conditions. For the moment, we propose the following recommendations for the use of LLMs to support review or editorial processes:

- Among other scholarly courses and modules such as responsible conduct of research, peer review trainings should educate researchers about the potential benefits and risks of using LLMs in review contexts. This should support researchers to use LLMs responsibly and make them aware of LLMs possible shortcomings and biases. As LLMs evolve quickly, such training should also be frequently updated.
- Content or supplementary documents of studies that contain sensitive information (e.g., health data) or protected data (e.g., anonymized interviews) should not be fed into LLMs unless security and data protection measures are put in place. Reviewers and editors who employ LLMs in review practices, should do so with due diligence. For example, in the case of ChatGPT, since it (currently) does not accept inputs as long as an entire manuscript’s length, one has to break down the content of a manuscript before feeding it to ChatGPT. For articles that have been published earlier and are included in the LLM’s training material, one can refer to the article without having to provide the full text (e.g., see our supplementary material), but this strategy does not work for newly published or unpublished manuscripts that have not been part of the LLM’s training material. In the latter case, the actual text has to be provided, potentially in multiple pieces. While there are confidentiality issues related to copy-pasting an unpublished manuscript into third-party platforms, to the extent that this might be non-problematic (e.g., feeding the introduction to see if authors have provided a good overview of a debate), one can imagine that a selective feeding of content, or not providing offered limitations or used references might result in biased or outright erroneous reviews. Indeed, LLMs are still in early stages of their development and for the moment seem only suitable to improve the first draft of a review instead of writing a review from scratch.
- Reviewers should disclose the use of LLMs and accept full responsibility for their reports’ accuracy, tone, reasoning and originality. Disclosures can be made in the beginning or end of the review reports as appropriate. Reviewers should specify whether they used LLMs and if so how, including details on 1) used prompt(s), 2) ideas or sections in the review report resulting from or affected by LLMs use, 3) the time and date of the use, and 4) parts of the manuscript that were fed into LLMs.
- Similarly, editors should adhere to full transparency regarding the use of LLMs or similar tools, either in the initial screening of manuscripts, the identification of reviewers, or the combining of review reports to come to final decisions.
- In adopting a precautionary approach, LLMs could integrate user monitoring systems to track abusive behavior and flag or report specific users. It should be noted that we recognize involved privacy concerns and believe that measures like this should be adopted cautiously and after careful deliberation.
- When LLMs are used in various review tasks, human agents should verify accuracy and take responsibility for their decisions and/or reports.
- Platforms that offer post-publication review services should indicate clearly how they expect their users to employ LLMs and under what conditions such use is considered appropriate. Furthermore, when these platforms employ LLMs themselves, this should be transparently disclosed.
- In encouraging various user groups to transparently disclose their use of LLMs, international committees and societies can play a significant role. For instance, the International Committee of Medical Journal Editors (ICMJE) can follow the Committee on Publication Ethics (COPE) that published a position statement [32], and besides taking a clear stance, encourage journal editors to develop specific policies and norms that fit their contexts.
- Finally, in light of all the uncertainties about the capabilities, limitations and inner-workings of LLMs, we encourage all user groups to keep experimenting with LLMs and to share findings and experiences. Such transparency about experiences with LLMs is crucial to enable a form of collective learning that allows the community to decide on the desirability and potential of LLMs usage in diverse contexts.

## Conclusion

We are likely at the very beginning of an era in which LLMs and future models will have a significant impact on many parts of society, including academia and scholarly communication. The question is therefore not *whether* these systems find their way to our daily practices of producing and reviewing scientific content, but *how* to use them responsibly. As sketched above, we believe that if used responsibly, LLMs have the potential to support publication and review practices. Uncertainties remain however, and various risks require us to engage with these systems with caution. Since this short essay has specific limitations (we only discussed review of journal articles and not other object types like grants, we used examples from ChatGPT, and were constrained by limitations of the used framework), we encourage commentary on this piece and advocate for wide community dialogue about the extent and ways that LLMs impact science and scholarship. In particular, the speed at which LLMs are being developed, requires continuous discussions about the implications of new models. Even in the relatively short time that this manuscript was under review, several new developments challenged some of the manuscript’s assertions. Among others, this includes the launch of GPT-4 as a successor of GPT-3.5 used in the examples in our manuscript. Such developments require the community to keep reflecting on the desirability, potential and risks of using LLMs in academic contexts.

## Supplementary Information

Additional file 1.

## Data Availability

Not applicable.
