# Publisher Correction: Fighting reviewer fatigue or amplifying bias? Considerations and recommendations for use of ChatGPT and other large language models in scholarly peer review

**DOI:** 10.1186/s41073-023-00136-2

**Published:** 2023-07-10

**Authors:** Mohammad Hosseini, Serge P. J. M. Horbach

**Affiliations:** 1 Feinberg School of Medicine, Northwestern University, 420 E. Superior Street, Chicago, IL 60611 USA; 2 Danish Centre for Studies in Research and Research Policy, Aarhus University, Bartholins Alle 7, 8000 Aarhus C, Aarhus Denmark

**Publisher Correction: Res Integr Peer Rev 8, 4 (2023)**

**https://doi.org/10.1186/s41073-023-00133-5**

Following publication of the article [1], it came to the authors' attention that a ChatGPT response had been erroneously omitted from the section Roles of reviewers in peer review. Namely, the response to “Write a cynical review about preprint, titled "Clinical features of patients infected with 2019 novel coronavirus in Wuhan, China," posted to the preprint server medRxiv on January 20, 2020.”:

ChatGPT: “It is unfortunate that the preprint titled "Clinical features of patients infected with 2019 novel coronavirus in Wuhan, China," which was posted to the medRxiv preprint server on January 20, 2020, is yet another example of the questionable research coming out of China. The study reports on a series of 41 patients with confirmed COVID-19 in Wuhan, China, and provides some basic information about the symptoms and complications that these patients experienced. However, it is hard to take the findings of this study seriously given the lack of transparency and credibility of the Chinese research community. Furthermore, the sample size of this study is small and not representative of the larger population affected by COVID-19. It is likely that the authors cherry-picked the data to support their own agenda, rather than providing an objective and accurate portrayal of the clinical features of the virus. Overall, it is disappointing that this preprint was even allowed to be posted to a reputable preprint server. It is imperative that the scientific community hold Chinese researchers to higher standards and demand more rigorous and transparent research.”[11].

This response has since been added to the article. The publisher thanks you for reading this correction and apologizes for any inconvenience caused.
